# Structure and enzymatic characterization of CelD endoglucanase from the anaerobic fungus *Piromyces finnis*

**DOI:** 10.1007/s00253-023-12684-0

**Published:** 2023-08-07

**Authors:** Alexey Dementiev, Stephen P. Lillington, Shiyan Jin, Youngchang Kim, Robert Jedrzejczak, Karolina Michalska, Andrzej Joachimiak, Michelle A. O’Malley

**Affiliations:** 1 Structural Biology Center, X-Ray Science Division, Argonne National Laboratory, Lemont, IL 60439 USA; 2 Department of Chemical Engineering, University of California, Santa Barbara, CA USA; 3 Department of Biochemistry and Molecular Biology, University of Chicago, Chicago, IL 60637 USA; 4 Biological Engineering Program, University of California, Santa Barbara, CA USA; 5 Joint BioEnergy Institute (JBEI), Emeryville, CA 94608 USA

**Keywords:** GH5 endoglucanase, Anaerobic fungi, Crystal structure, Enzyme kinetics, Lignocellulose

## Abstract

**Abstract:**

Anaerobic fungi found in the guts of large herbivores are prolific biomass degraders whose genomes harbor a wealth of carbohydrate-active enzymes (CAZymes), of which only a handful are structurally or biochemically characterized. Here, we report the structure and kinetic rate parameters for a glycoside hydrolase (GH) family 5 subfamily 4 enzyme (CelD) from *Piromyces finnis*, a modular, cellulosome-incorporated endoglucanase that possesses three GH5 domains followed by two C-terminal fungal dockerin domains (double dockerin). We present the crystal structures of an apo wild-type CelD GH5 catalytic domain and its inactive E154A mutant in complex with cellotriose at 2.5 and 1.8 Å resolution, respectively, finding the CelD GH5 catalytic domain adopts the (β/α)_8_-barrel fold common to many GH5 enzymes. Structural superimposition of the apo wild-type structure with the E154A mutant-cellotriose complex supports a catalytic mechanism in which the E154 carboxylate side chain acts as an acid/base and E278 acts as a complementary nucleophile. Further analysis of the cellotriose binding pocket highlights a binding groove lined with conserved aromatic amino acids that when docked with larger cellulose oligomers is capable of binding seven glucose units and accommodating branched glucan substrates. Activity analyses confirm *P. finnis* CelD can hydrolyze mixed linkage glucan and xyloglucan, as well as carboxymethylcellulose (CMC). Measured kinetic parameters show the *P. finnis* CelD GH5 catalytic domain has CMC endoglucanase activity comparable to other fungal endoglucanases with *k*_cat_ = 6.0 ± 0.6 s^−1^ and *K*_m_ = 7.6 ± 2.1 g/L CMC. Enzyme kinetics were unperturbed by the addition or removal of the native C-terminal dockerin domains as well as the addition of a non-native N-terminal dockerin, suggesting strict modularity among the domains of CelD.

**Key points:**

• *Anaerobic fungi host a wealth of industrially useful enzymes but are understudied*.

• *P. finnis CelD has endoglucanase activity and structure common to GH5_4 enzymes*.

• *CelD’s kinetics do not change with domain fusion, exhibiting high modularity*.

**Supplementary Information:**

The online version contains supplementary material available at 10.1007/s00253-023-12684-0.

## Introduction

Microbial degradation of lignocellulose is a fundamental biological process that is crucial to nutrient cycling in nature, a process that turns over ~ 10^15^ pounds of plant biomass and releases the equivalent amount of energy in 640 billion barrels of oil per year (Boudet et al. 2003; Ragauskas et al. 2006). Microbes achieve such efficient degradation of lignocellulose by employing a synergistic consortium of enzymes that attack the different biopolymers present in plant cell walls—hemicellulose, lignin, and most abundantly, cellulose. The core synergism promoting cellulose saccharification occurs among various carbohydrate-active enzymes (CAZymes) of the glycoside hydrolase (GH) family, including endo-cleaving enzymes, which randomly cleave internal β-1,4 linkages, cellobiohydrolases that progressively remove cellobiose units from chain ends, and β-glucosidases which hydrolyze the freed cellobiose into glucose (Woodward 1991; Wilson and Wood 1992). The complete depolymerization of hemicellulose is achieved in a similar manner, though a greater diversity of GH family enzymes, as well as enzymes from other families including carbohydrate esterases (CE), are required to break the diverse sugar linkages and branched chains present in hemicellulose (Lowe et al. 1987; Mountfort and Asher 1989; Moreira and Filho 2008, 2016). More recently, the importance of auxiliary, non-hydrolytic enzymes, such as lytic polysaccharide monooxygenases (LPMOs), to synergistic lignocellulose degradation has also been emphasized (Hemsworth et al. 2015; Forsberg et al. 2019).

While this enzyme consortium consists exclusively of freely diffusing enzymes in aerobic microorganisms, anaerobic bacteria and fungi have evolved multi-enzyme complexes called cellulosomes that colocalize lignocellulolytic enzymes to greatly enhance their degradative activity (Dijkerman et al. 1997b; Artzi et al. 2017). As such, cellulosomes make attractive potential components of enzyme cocktails for industrial waste biomass valorization. In both bacterial and fungal systems, noncatalytic dockerin domains on the C- and/or N-termini of proteins mediate complex formation via interaction with cohesin repeat domains on a central scaffolding protein, though these domains share no sequence homology between the two systems (Fontes and Gilbert 2010; Haitjema et al. 2017). Bacterial cellulosomes from several different species of anaerobic bacteria have been studied extensively for over three decades and the structures and mechanisms of the dockerin-cohesin interaction are well understood, enabling the construction of engineered cellulosomes optimized for lignocellulose saccharification (Carvalho et al. 2003; Adams et al. 2006). Despite knowledge that fungal cellulosomes harbor greater enzyme diversity to degrade more recalcitrant biomass (Lankiewicz et al. 2023) and produce glucose rather than cellobiose as the major degradation product (Trinci et al. 1994; Solomon et al. 2016), a mechanistic understanding of fungal cellulosome assembly remains incomplete. The fungal dockerin, originally annotated as a carbohydrate-binding module (CBM) family 10 due to its sequence homology to the CBM10 protein family (InterPro family IPR002883), was first identified as a modular protein binding domain in the 1990s (Fanutti et al. 1995). Only two structures of fungal dockerin domains exist (Raghothama et al. 2001; Nagy et al. 2007) and the identity and structure of the companion cohesin domain to which they bind remain unknown, with many potential candidates suggested over the last several decades (Steenbakkers et al. 2001; Gilmore et al. 2015; Haitjema et al. 2017).

A rich catalog of past literature on anaerobic fungi supports their potential for exploitation in industrial bioprocessing (Akin et al. 1990; Dijkerman et al. 1997a; Solomon et al. 2016). Anaerobic fungi are prolific producers of CAZymes, encoding on average over four-fold more CAZymes than *Trichoderma reesei* and *Aspergillus niger*, the sources of the most popular cellulolytic cocktails (www.mycocosm.jgi.doe.gov; Seppälä et al. 2017). In addition to encoding many cellulosome-incorporating CAZymes, anaerobic fungal genomes encode many modular proteins with multiple enzymatic activities, which is rare among lignocellulolytic organism genomes sequenced to date (Brunecky et al. 2013; Cragg et al. 2015; Jia and Han 2019). Furthermore, the secreted proteome has been shown to rival and even exceed the cellulolytic activity of *T. reesei* and significantly exceed the hemicellulose degrading power of traditional enzyme cocktails (Wood et al. 1986; Solomon et al. 2016). Thus, mining anaerobic fungal genomes for better lignocellulolytic enzymes may yield higher performing enzymes for industrial biomass valorization applications.

However, only seven unique enzymes from anaerobic fungi have crystal structures (PDB IDs: 6IDW, 5YN3, 5U22, 5CXU, 3WP4, 3AYR, 2C1F), presenting a challenge to understanding what structural characteristics impart high lignocellulolytic activity to these organisms. Fungal cellulosomes are particularly interesting due to their robust hydrolytic activity, but previous works measuring positive or negative contributions to enzyme activity by dockerin domains have produced conflicting results, obfuscating whether dockerin fusion and cellulosome incorporation alter the kinetics of individual enzymes (Fanutti et al. 1995; Huang et al. 2005; Gilmore et al. 2020). These knowledge gaps in the biochemistry of individual anaerobic fungal CAZymes themselves, and of fungal cellulosomes comprising many CAZymes, present a challenge towards designing lignocellulolytic enzyme cocktails that leverage the degradative machinery of anaerobic fungi.

In this study, we characterized, at the structural and functional levels, the CelD enzyme from the anaerobic fungus *Piromyces finnis*. This fungal enzyme belongs to the glycoside hydrolase (GH) family 5 subfamily 4 of cellulases and acts strictly as an endo-β-1,4-glucanase (EC 3.2.1.4) with confirmed activity against carboxymethylcellulose (CMC), mixed linkage glucan (MLG), and xyloglucan. The full-length CelD is a 136 kDa multi-domain enzyme containing three GH5 domains and two C-terminal dockerin domains, also termed a “double dockerin” (GenBank ORX48147.1). To facilitate bacterial protein expression and to study the contribution of fungal dockerins to enzyme catalytic activity, we characterized a single GH5 catalytic domain in isolation (ORX48147.1 residues 748–1105), as well as a GH5 with the native C-terminal double dockerin (ORX48147.1 residues 748–1192) and a GH5 with native C-terminal double dockerin and a non-native N-terminal dockerin domain. We present the crystal structure of a CelD GH5 catalytic domain in its apo wild-type form and complementary structure of an inactive E154A mutant in complex with cellotriose solved at a resolution of 2.5 Å and 1.8 Å, respectively (for convenience, in this work, we used 1–362 numbering for the catalytic domain which corresponds to 91–452 residue numbering of the “1–536” in Supplemental Table S1). We also measure kinetic parameters for CMC hydrolysis of three CelD constructs with and without dockerin domains. CelD possesses kinetic parameters comparable to other fungal endoglucanases in rate constant and catalytic efficiency. We observe no change in these parameters upon the addition of natural and unnatural dockerin domain fusions, indicating CelD’s catalytic and dockerin domains are highly modular and that this enzyme’s performance does not change when incorporated into a fungal cellulosome. Overall, the presented atomic-resolution structure and detailed biochemical characterization of CelD add to our growing understanding of how anaerobic fungal cellulosomes rapidly degrade biomass and provide additional insight towards leveraging anaerobic fungal enzymes for industrial biomass valorization applications.

## Methods

### Enzyme cloning, expression, and purification

The cDNA fragment corresponding to the cellulase catalytic domain CelD (residues 748–1105, GenBank accession number: ORX48147.1) was cloned from the anaerobic fungus *P. finnis* isolated from horse feces (Solomon et al. 2016; Haitjema et al. 2017). The PCR amplified construct (denoted 91–452) was cloned into pMCSG68 expression vector with the sequence for an N-terminal His-tag of MHHHHHHSSGVDLWSHPQFEKGTENLYFQSNA (Kim et al. 2011). cDNA for construct 91–536, containing the third CelD GH5 domain with native C-terminal double dockerin (residues 748–1192, GenBank accession number: ORX48147.1), was obtained as described above. Construct 1–536 was obtained by cloning a non-native, N-terminal dockerin domain from *P. finnis* upstream of construct 91–536 in the pMCSG68 backbone. The E154A point mutation within construct 91–452 was introduced using a QuickChange kit (Agilent, Santa Clara, CA, USA) according to the manufacturer’s protocol using the following primers: 5′-GGTCAAAACGCACCAAGAAAGAACGGTACTCCAGTTGA-3′ as a forward primer and 5′-CTTTCTTGGTGCGTTTTGACCTTCGAAGATTAAACGTTCA-3′ as a reverse primer. Both wild-type and mutated amino acid sequences were verified by nucleotide sequencing of the cloned constructs.

Recombinant catalytic domains of wild-type CelD with and without dockerin domain fusions as well as the inactive E154A mutant were expressed in *Escherichia coli* BL21-gold (DE3) (New England Biolabs, Ipswich, MA, USA) cells by induction with 0.5 mM isopropyl *β*-D-1-thiogalactopyranoside at 18 °C for 12 h. Following induction, cells were lysed and the 6 × His-tagged proteins purified from soluble cell lysate using a nickel-nitrilotriacetic acid HisTrap column (Cytiva Life Sciences, Marlborough, MA, USA) on an AKTA express system (Cytiva Life Sciences, Marlborough, MA, USA). Protein purity was confirmed by routine SDS-PAGE analysis. Native PAGE analysis revealed the presence of multimers for dockerin-containing constructs (data not shown).

After routine immobilized metal affinity chromatography, protein for crystallization then underwent proteolytic cleavage by a TEV protease at 4 °C for 12 h. Uncleaved protein and His-tagged TEV protease were removed by AKTA express using a HisTrap column (Cytiva Life Sciences, Marlborough, MA, USA) to remove uncleaved product and His-tagged TEV protease. Cleaved CelD catalytic domain or the E154A mutant was then isolated by size exclusion chromatography on a Superdex 200 column (Cytiva Life Sciences, Marlborough, MA, USA) in 15 mM Tris–HCl buffer, pH 7.5, supplemented with 150 mM NaCl.

### Complex formation, crystallization, and structure determination

The best crystals of apo wild-type of the catalytic domain were obtained at 16 °C from sitting drops containing 0.4 µL of the protein sample at concentration of 21 mg/mL and 0.4 µL of reservoir solution consisting of 0.1 M Tris–HCl buffer, pH 8.7, 0.5 M LiCl_2_, and 28% PEG 6000. The complex between the E154A mutant protein and cellotriose was formed by adding the ligand stock solution (Sigma-Aldrich, St. Louis, MO, USA) to the protein solution at 10 mM final concentration. The complex crystals were produced using a similar crystallization approach to that described above except the reservoir solution contained 0.1 M sodium acetate, 1.2 M LiCl_2_, and 24% PEG 6000, and the protein concentration was 16 mg/mL. Several cycles of microseeding under similar crystallization conditions were carried out to obtain crystals suitable for X-ray analysis for both structures. For data collection, crystals were harvested with 20% (v/v) ethylene glycol in the reservoir solution. Diffraction data were collected from a single flash-frozen crystal on the Structural Biology Center beamlines, 19-ID for the apo form and 19-BM for the E154A mutant-cellotriose complex (Advanced Photon Source, Argonne National Laboratory, Lemont, IL, USA). Data were indexed and processed with HKL-3000 software suite (Otwinowski and Minor 1997).

The structure of the apo form of the catalytic domain was solved by molecular replacement using the molrep program (Vagin and Teplyakov 1997) from the HKL3000 software suite, with the structure of the catalytic domain of EglA GH5 endoglucanase from *Piromyces rhizinflata* (PDB code 3AYR) as a search model (McCoy et al. 2007). The refined model of the apo form structure was used as a search model to solve the structure of the complex between the E154A mutant and cellotriose. The final refined models of both structures were obtained by carrying out several iterative alternative cycles consisting of manual model building using COOT (Emsley and Cowtan 2004) and phenix.refine (Adams et al. 2010) until the model converged to the stereochemically good models with *R*_work_/*R*_free_ of 0.188/0.230 for the apo form and 0.176/0.205 for the E154A mutant-cellotriose complex as indicated in Table 1. Both structures were validated by Ramachandran plot and MolProbity (Laskowski et al. 1993; Chen et al. 2010) and RCSB validation before the coordinates were deposited in the Protein Data Bank (PDB codes 8GHX and 8GHY for the apo and the E154A mutant complex, respectively).

**Table 1:** Data collection and refinement statistics for crystal structure determination

	Apo wild-type CelD	E154A CelD-cellotriose complex
Data collection		
Wavelength (Å)	0.9792	0.9792
Resolution range (Å)	50.00–2.45 (2.49–2.45)^a^	50.00–1.80 (1.83–1.80)
Space group	P2_1_2_1_2_1_	P2_1_2_1_2_1_
Unit cell (*a*, *b*, *c*) (Å)	69.726, 81.850, 133.259	69.495, 81.007, 131.922
Total reflections	296,064	421,385
Unique reflections	28,052 (1348)	69,108 (3429)
Multiplicity	10.6 (6.4)	6.1 (5.9)
Completeness (%)	99.2 (98.3)	98.9 (99.2)
< *I* > /σ (*I*)	14.5 (2.1)	21.2 (1.4)
* R*_merge_^b^	0.044 (1.741)	0.1000 (1.578)
Refinement		
Resolution range (Å)	49.36–2.46 (2.55–2.46)	30.00–1.80 (1.82–1.80)
* R*_work_/*R*_free_^c^ (%)	18.8 (23.5)/23.0 (28.2)	17.6 (31.2)/20.4 (35.3)
Number of non-hydrogen atoms^d^	5918	6391
Protein	5784	5834
Ligands	40	68
Waters	94	489
Clash score	3.08	1.56
Rotamer outlier (%)	1.84	1.48
RMS (bonds, Å)	0.002	0.004
RMS (angles, ^°^)	0.413	0.658
Ramachandran favored (%)	95.36	95.83
Ramachandran allowed (%)	4.5	4.17
Ramachandran outliers (%)	0.14	0.0
Average B-factor (Å^2^)	44.3	34.2
Macromolecules	44.4	33.8
Ligands	47.5	34.2
Waters	37.7	38.4
PDB code	8HGX	8HGY

^a^Statistics for the highest-resolution shell are shown in parentheses

^b^*R*_merge_ = 100Σ(*h*)Σ(*i*)|*I*(*i*)- < *I* >|/ Σ(*h*)Σ(*i*)*I*(*i*), where *I*(i) is the *i*th intensity measurement of reflection *h*, and < *I* > is the average intensity from multiple observations

^c^*R* = Σ||*Fobs*|-|*Fcalc*||/ Σ|*Fobs*|. Where *Fobs* and *Fcalc* are the structure factor amplitudes from the data and the model, respectively. 5% reflections were used to calculate *R*_free_ values and were omitted from the structure refinement and *R*_W_ calculation

^d^Per asymmetric unit

### Enzymatic activity assays

Cellulolytic activity of *P. finnis* CelD on CMC, beechwood xylan, MLG, xyloglucan, phosphoric acid swollen cellulose (PASC), and arabinogalactan was assessed using the dinitrosalicylic acid (DNS) reducing sugar assay essentially as described elsewhere (King et al. 2009). CMC was purchased from Sigma-Aldrich (St. Louis, MO, USA); xyloglucan, MLG, and arabinogalactan were purchased from Fisher Scientific (Waltham, MA, USA); and beechwood xylan was purchased from Megazyme (Bray, Ireland). PASC was prepared as described previously (Morag (Morgenstern) et al. 1992). For specific activity measurements on CMC, MLG, PASC, and xyloglucan, 200-µL reactions in 0.1 M sodium acetate pH 5.5 containing CelD at 0.70 µM and substrate at 1% w/v final concentration were statically incubated at 39 °C. The same mixture substituting enzyme for acetate buffer was used as a negative control. Three 60-µL samples were taken after 1, 2, and 24 h of incubation time and their reducing sugar composition was measured using the DNS assay (King et al. 2009). Briefly, 100 µL of DNS was added to each reaction sample and the mixture is incubated at 95 °C for 5 min. One hundred microliters of this mixture was added to 100 µL of water and the absorbance at 540 nm was measured using a Tecan Infinite® M1000 plate reader (Tecan Group, Männedorf, Switzerland). *A*_540nm_ was converted to g/L glucose equivalents with a standard curve of glucose in 0.1 M sodium acetate buffer pH 5.5. Specific activities were calculated using protein concentrations measured by *A*_280nm_ with appropriate parameters and reducing sugar concentrations measured by DNS assay using standard curves of glucose. Absorbance measurements were blank subtracted by a negative control of substrate without enzyme.

For specific activity measurements on xylan and arabinogalactan, 30 µL of protein in 0.1 M sodium acetate pH 5.5 (0.1 mg total protein) was added to 30 µL of 2% (w/v) freshly prepared, unautoclaved polysaccharide solution in 0.1 M sodium acetate (pH 5.5). Reactions were performed at 39 °C unless otherwise stated and in triplicate, with reaction times of 45 min for xylan and 14 h for arabinogalactan.

Kinetic parameters for CelD on CMC were obtained by adding 5 µL enzyme in 0.1 M sodium acetate pH 5.5 to 195 µL of pre-warmed CMC substrate at concentrations of 0–30 g/L CMC to a final enzyme concentration of 0.05 µM. Enzyme–substrate mixtures were incubated at 39 °C with shaking at 188 RPM. Three 60-µL samples were taken after 5–35 min of incubation time, and their reducing sugar composition was measured as described above. Initial rates were extracted by linear regression of the *A*_540nm_ vs time curve and converted to the appropriate units using a glucose standard curve after subtraction of *A*_540nm_ signal from a substrate only control. Initial rate vs substrate concentration data were then fit to a Michaelis–Menten model by nonlinear regression using the SciPy Python package (https://scipy.org/) to determine *k*_cat_ and *K*_m_ parameters for each GH5 variant.β-Glucosidase and β-galactosidase activities were assessed by adding 30 µL of protein (0.1 mg total protein) to 970 µL of 5 mM 4-nitrophenyl β-D-glucopyranoside (*p*NPG) or 4-nitrophenyl β-D-galactopyranoside (*p*NPGal) in 50 mM sodium phosphate (dibasic) buffer (pH 7.0) with 2% (w/v) bovine serum albumin. Absorbance at 405 nm as measured by a Tecan Infinite® M1000 plate reader tracked reaction progression over 24 h.

## Results

### Overall structure of the CelD catalytic domain

**Fig. 1:**
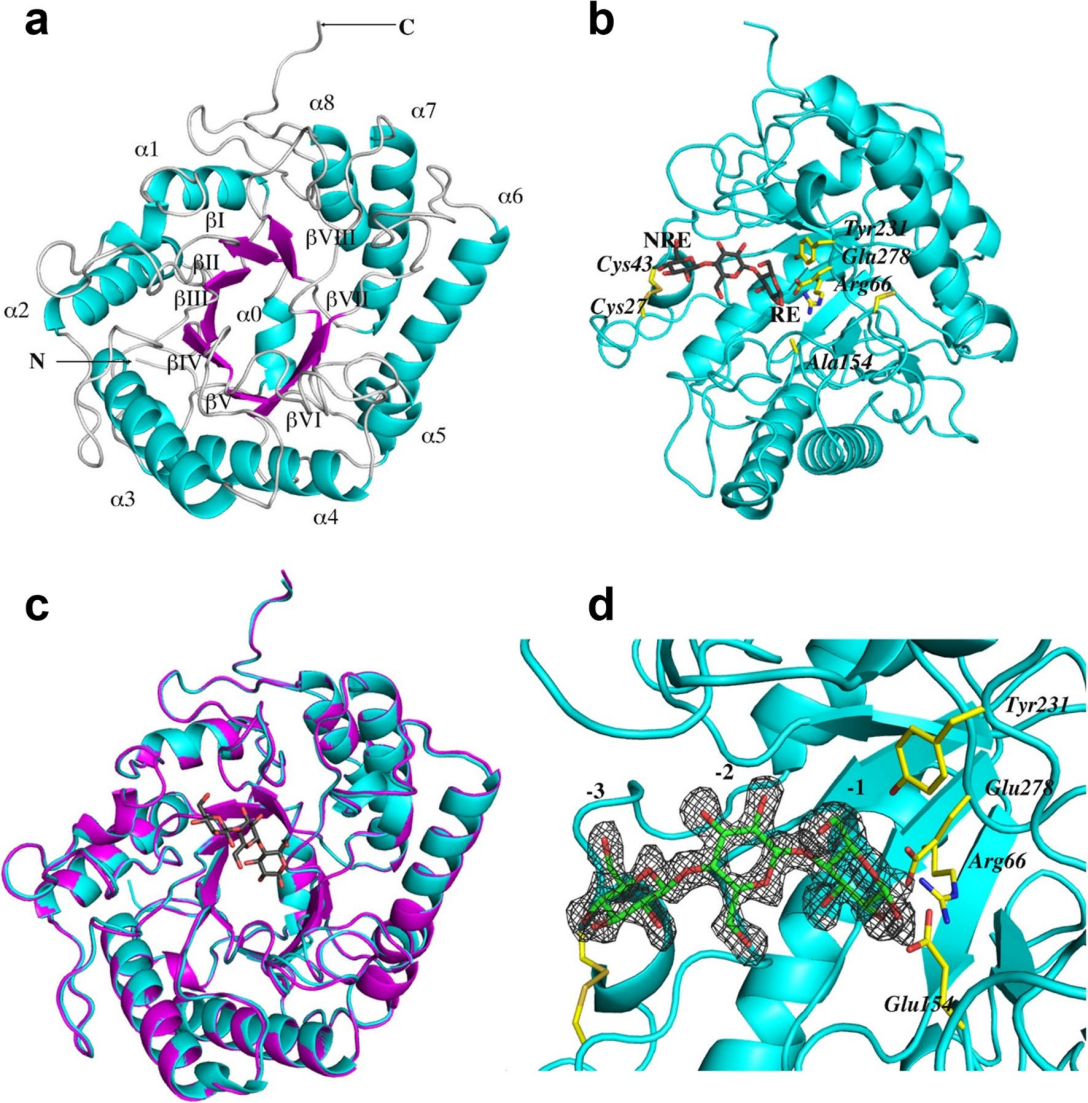
The overall structure of the CelD catalytic domain. **a** Ribbon diagram of the apo-CelD structure with the secondary structural elements indicated. The α-helixes (α1–α8, cyan) flanking the β-strands (βI–βVIII, magenta) are labeled. Arrows indicate the N- and C-termini of the protein. **b** The structure of the E154A CelD variant in complex with the cellotriose (gray sticks). One disulfide bond and the catalytic residues are indicated and are shown as a yellow stick model. The reducing (RE) and non-reducing (NRE) ends of the oligosaccharide are indicated. **c** Superposition of the apo wild-type (magenta) and the E154A-ligand (cyan) crystal structures. The catalytic domains are superimposed by aligning the Cα atoms and are presented as ribbon diagrams. The cellotriose ligand is shown as a gray stick. **d** The substrate binding area in the ligand-bound complex. The protein moiety is presented as a cyan ribbon. The cellotriose molecule (green) bound to the − 3, − 2, and − 1 glucose-binding subsites and the residues of the active site (yellow) are shown as sticks. The electron density map (gray mash) around the bound ligand is countered at the 1.4σ level

The unliganded structures of the wild-type catalytic domain and its inactive E154A mutant in complex with cellotriose were determined by molecular replacement and refined to 2.5 Å and 1.8 Å resolution, respectively (Table 1 and Fig. 1). The crystals of the apo form and the complex belonged to orthorhombic P2_1_2_1_2_1_ space group and contained two domain molecules per asymmetric unit. Almost all amino acid residues of the CelD catalytic domain, with the exception of a few side chains and three C-terminal residues, were traceable in the final electron density map. The overall root mean square deviation (RMSD) between apo wild form and inactive E154A-ligand complex protein models was 0.26 Å for 359/362 Cα pairs, demonstrating high overall similarity and illustrating that substrate binding results in little to no conformational change (Fig. 1). The CelD catalytic domain displays strong structural similarity to GH clan A, a group of 28 unique GH families exhibiting a (β/α)_8_–barrel fold in structure, with the highest sequence similarity to proteins from GH5 subfamily 4 (GH5_4), a family of enzymes that predominantly display endoglucanase activity (EC 3.2.1.4) (Fig. 1a) (Jenkins et al. 1995; Pickersgill et al. 1998; Drula et al. 2022). In addition to the eight core β/α elements, CelD has another three small helixes located between *α*_4_/*β*_V_, *β*_V_/*α*_5_, and *β*_VI_/*α*_6_ secondary structure elements and two short β-strands located on the loop between C-terminal *β*_VIII_/*α*_8_ elements (Supplemental Fig. S1).

The sequences most homologous to *P. finnis* CelD that have solved experimental structures were all GH5_4 enzymes from *P. rhizinflata* (PDB: 3AYR, 82% identity), *Ruminococcus champanellensis* (PDB: 6WQP, 43.1% identity), *Acetivibrio cellulolyticus* (PDB: 6MQ4, 39.4% identity), and *Clostridium cellulovorans* (PDB: 3NDY, 40% identity) (Tseng et al. 2011; Glasgow et al. 2020) (Supplemental Fig. S1). These structures align to *P. finnis* CelD with Cα RMSDs of 0.45 Å, 1.37 Å, 1.44 Å, and 1.82 Å for 3AYR, 6WQP, 6MQ4, and 3NDZ respectively. All enzymes conserve the catalytic glutamic acid residues at positions 154 and 278 characteristic of GH5 enzymes (Jenkins et al. 1995) and many of the strictly conserved sites in the multiple sequence alignment (MSA) encode aromatic amino acids, suggesting their role in substrate binding. The primary structural feature differentiating the apo structures is the loop connecting the *β*_1_-strand with the *α*_1_-helix (Fig. 1a and Supplemental Fig. S1). CelD and 3AYR both have 13 residue loops pinned by a disulfide bond between Cys27 and Cys43. 6WQP maintains a long loop of 12 residues, while 6MQ4 and 3NDY contain shorter loops of 9 and 2 residues respectively. Other loops exhibiting structural diversity among the enzymes connect the *β*_6_-strand with the *α*_6_-helix and the *β*_8_-strand with the *β*_9_-strand (Supplemental Fig. S2). The proximity of these loops to the substrate binding site (Fig. 1b) suggests their structure, and amino acid composition plays an important role in substrate binding specificity.

As only nine GH5 structures from the fungal kingdom have been solved to date, we sought to compare *P. finnis* CelD to other fungal GH5 structures. Based on a sequence alignment of CelD with three fungal GH5 members studied at the structural level (*Piromyces rhizinflata* EglA (PDB 3AYR), a GH5_4; *Trichoderma reesei* EgII (PDB 3QR3), a GH5_5; and *Thermoascus aurantiacus* EngI (PDB 1GZJ), a GH5_5), EglA from *P. rhizinflata* is unsurprisingly the closest structural homolog from the fungal kingdom to CelD with sequence identity to CelD of 82% (Supplemental Fig. S3) (Lo Leggio and Larsen 2002; Lee et al. 2011; Tseng et al. 2011). Optimal superposition of the CelD catalytic domain structure with corresponding EglA, EgII, and EngI homologous domains results in 356, 242,, and 254 equivalent Cα atoms with the RMSD values of 0.43 Å, 1.91 Å, and 2.22 Å, respectively (Supplemental Fig. S4). Although these enzymes all share the same basic (*β*/*α*)_8_–barrel topology, they exhibit significant sequence diversity in the loop areas connecting the major structural elements. There are several extra residue insertions observed for CelD and EglA in the loops between *β*_I_/*α*_1_, *β*_IV_/*α*_4_, *α*_5_/*β*_VI_, and *β*_VIII_/*α*_8_ compared to very compact loop structures of EgII and EngI. As previously mentioned for the N-terminal loop, these loops could contribute differently to substrate binding and enzyme specificity as well as to thermal stability of the enzyme. It is also interesting to note that the *T. reesei* EgII cellulase contains 8 cysteine residues, which form four disulfide bonds, and one of these bridges (Cys222–Cys249), pinning the *α*_6_/*β*_VI_ loop to the N-terminus of the α_7_-helix, corresponds to that observed in *Thermoascus aurantiacus* EngI (Supplemental Fig. S3 and S4). *T. aurantiacus* EngI is a hyperthermophilic enzyme with a *T*_*m*_ of about 81 °C; meanwhile, the reported *T*_*m*_ value for *T. reesei* EgII is 69.5 °C (Lee et al. 2002). CelD and EglA also exhibit one disulfide bridge located in the N-terminal loop, but there is no thermal stability data yet available for these enzymes.

### Structure of the CelD-cellotriose complex

**Fig. 2:**
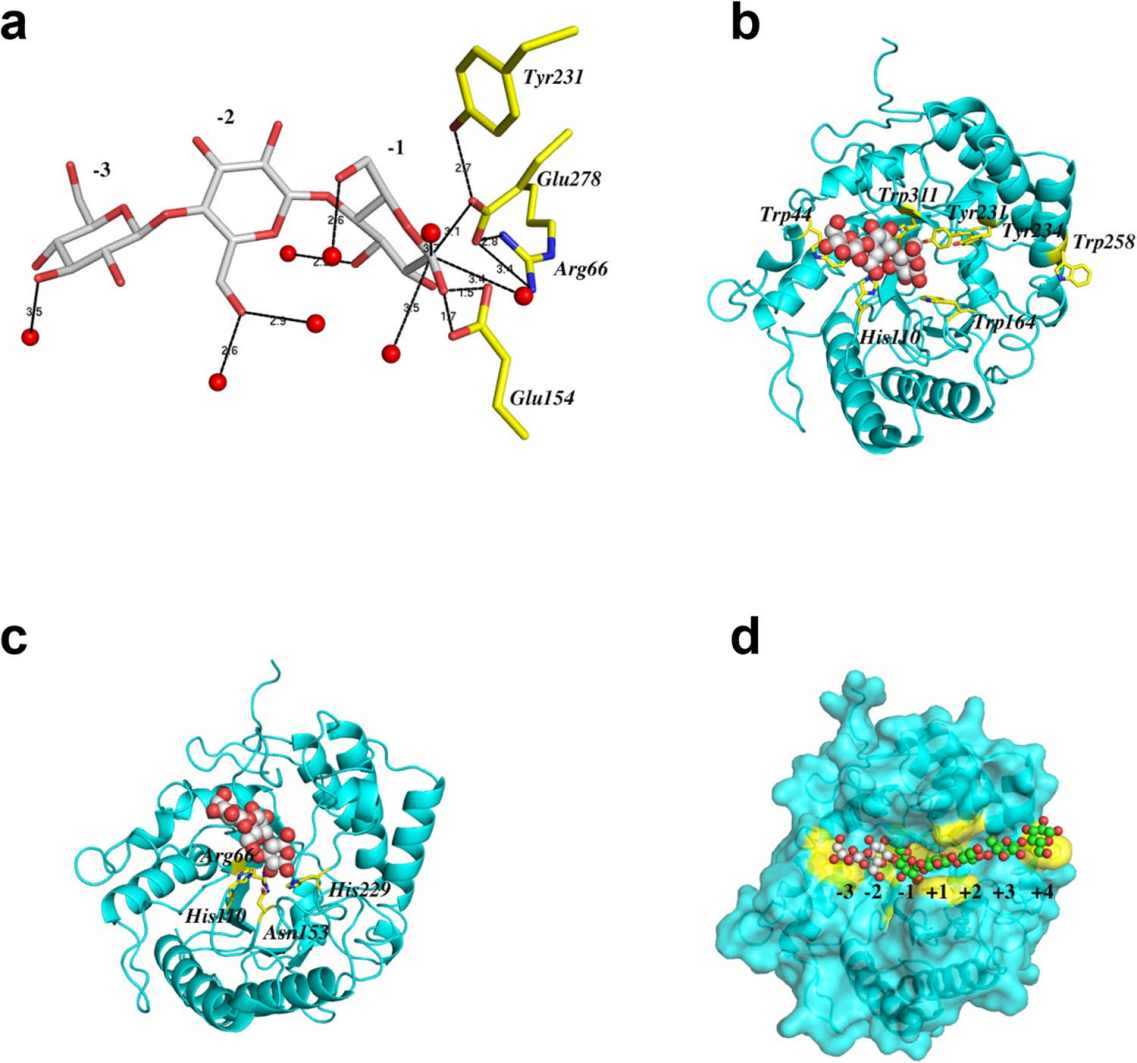
Substrate binding area of the CelD cellulase. **a** Zoomed in view of the active site with bound cellotriose molecule (gray stick) surrounded by 8 water molecules (red spheres) and several catalytic residues (yellow stick). The hydrogen bonds are shown as black dotted lines, and the locations of the substrate binding sites (the − 3, − 2, and − 1) are labeled. **b** Ribbon representation of the CelD-ligand complex (cyan). The positions of the conserved aromatic residues involved in substrate binding are shown in yellow stick; the cellotriose ligand is shown as gray spheres. **c** The same as in **b** except that the positions of the conserved and spatially conserved residues associated with cellulolytic activity are shown in yellow stick. **d** Total view of the protein surface (in the same color as in **b** and **c**) showing the wide CelD active side cleft with the modeled hepta-oligosaccharide substrate is presented. The hepta-oligosaccharide is shown as spheres. The modeled saccharide units from the − 1 subsite to the + 4 subsite are shown as green spheres, and experimentally observed carbohydrate units at the − 3 and − 2 subsites are shown as gray spheres. The scissile glycoside bond is between the − 1 and + 1 sites. The positions of conserved aromatic residues served to mediate carbohydrate binding in the encounter complex are indicated in yellow color

Attempts to crystallize the CelD active catalytic domain with cellobiose or cellotriose substrates were unsuccessful using both soaking and co-crystallization approaches. We additionally attempted to crystallize CelD with its natural dockerin domains, which would have represented the first full structure of a fungal dockerin-fused enzyme but failed to get high-quality crystals. After introducing the inactivating E154A mutation to the active site of a single CelD catalytic domain, we were able to obtain high-quality crystals of the E154A-cellotriose complex. As described above, we did not observe substantial conformational changes between the apo-enzyme structure and the mutant structure with the bound ligand. Thus, the catalytic residues in the apo-CelD structure are likely to be in a catalytically competent position and superposition of the active enzyme structure and the mutant structure with the bound ligand provides sound structural information about the substrate binding and mechanism of the enzyme catalysis. Like other GH5 enzymes, CelD contains two invariant catalytic glutamate residues, the acid/base Glu154 and the nucleophile Glu278. Superposition of two structures solved in this work confirms that the carboxylate OE1 and OE2 oxygens of the catalytic acid/base glutamate point towards the O1 atom of the − 1 glucopyranose unit at 1.5 Å and 1.7 Å, respectively, and the carboxylate oxygen of the complementary nucleophile glutamate forms a hydrogen bond with the anomeric C1 carbon of the same saccharide unit at 3.1 Å (Fig. 2a). Meanwhile, the nucleophile Glu278 is sandwiched between two conserved Arg66 and Tyr231 residues which form hydrogen bonds to the carboxylate oxygens and appear to serve a supportive role to stabilize this Glu residue throughout catalysis as observed for other GH5 enzymes (Fig. 2c) (Dominguez et al. 1995; Tseng et al. 2011).

We found 8 ordered water molecules making multiple hydrogen bonds with both the protein moiety and the cellotriose molecule in the ligand-bound structure; two of them are located near the anomeric carbon of the − 1 saccharide moiety (Fig. 2a). One of these water molecules may participate in the catalytic mechanism of the CelD as a nucleophilic attack on the glycosyl-enzyme intermediate (Davies et al. 1998).

### Holo CelD E154A-Cellotriose structure reveals key substrate recognition sites

Structural comparison of the CelD cellulase with other known GH5_4 family enzymes suggests that a cleft containing the enzyme active site may provide a favorable platform for binding oligomers up to seven sugar units within the − 3 to + 4 subsites. This cleft presents a flat platform for interacting with negative sugar subsites and a U-shaped groove that appears to orient the substrate for catalysis and provide interaction sites for positive sugar subsites. The CelD active site groove is lined with aromatic residues, Trp44, Trp164, Tyr231, Tyr234, Trp258, and Trp311, and all these positions except Trp258 are strictly conserved among the GH5_4 enzymes we analyzed (Supplemental Fig. S1). Trp164 and Tyr234 are proximal to the + 2 and + 3 subsites and Trp44 and Tyr231 are close to the − 3 and − 1 binding sites, respectively (Fig. 2b, d). Trp258 is positioned to interact with a linear polysaccharide chain at the + 4 position.

**Fig. 3:**
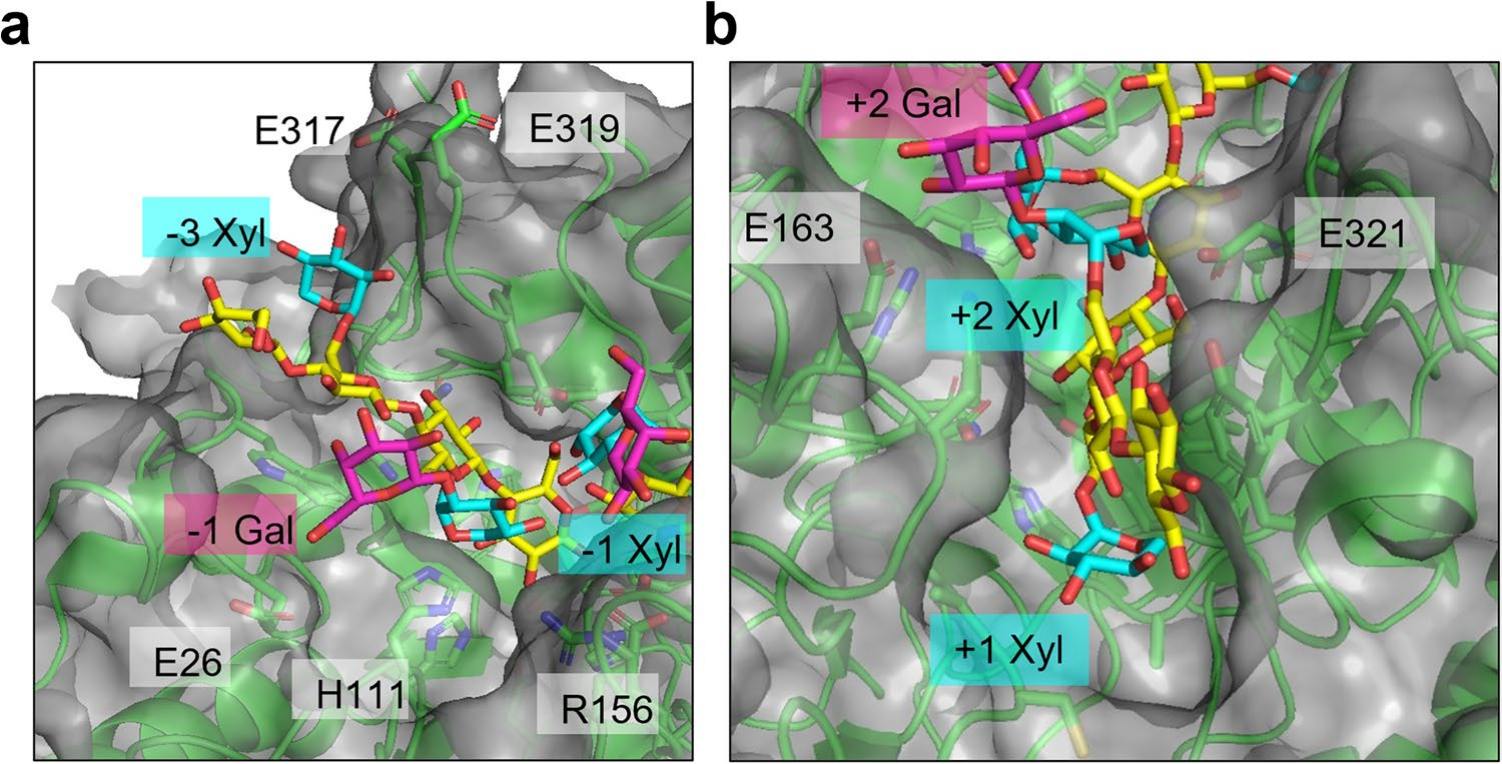
Potential contacts between xyloglucan branched sugars and CelD modeled by alignment with 4W88 and 2JEQ with annotated CelD residues interacting with negative substrate subsites (**a**) and positive substrate subsites (**b**). CelD is shown both as a gray surface and green cartoon representation to visualize both atomic contacts and the fit of xyloglucan to the active site

The overall shape of the CelD polysaccharide binding site appears optimal for strictly linear polysaccharides, but an indent in the enzyme surface into which the C6 atom of the − 2 backbone glucose points (Fig. 2c) suggests this enzyme may accommodate a substrate like xyloglucan, which contains a glucose backbone with branched xylose and galactose sugars. Structural and biochemical analysis of several GH5_4 enzymes suggests some structural and sequence signatures indicative of enzyme activity on branched polysaccharide substrates include aromatic and polar side chains within the loops between *β*_3_ and *α*_4_, *β*_4_ and *α*_5_, and *β*_8_ and *β*_9_ (Glasgow et al. 2020). Aligning the CelD structure to the xyloglucan oligosaccharide-bound structures 2JEQ (Gloster et al. 2007) and 4W88 (dos Santos et al. 2015) suggests CelD can spatially accommodate xyloglucan, with potential favorable hydrogen bonding interactions with the − 3 xylose branch at E317 or E319; the − 2 xylose and galactose involving H111, R156, and E26; the + 2 galactose at E163; and the + 2 xylose at E321 (Fig. 3). However, CelD does not appear to have any aromatic side chains poised to interact with branched sugars that would suggest this enzyme is specific for branched polysaccharides.

### Characterization CelD substrate specificity and enzyme kinetics

We tested the *P. finnis* CelD catalytic domain against several soluble and insoluble substrates to determine the catalytic domain’s specificity for cellulose vs hemicellulose polysaccharides, its endoglucanase vs β-glucosidase activity, and its preference for β-1,4 vs other α linkages. As indicated by structural analysis, CelD hydrolyzes the linear cellulose analog carboxymethylcellulose (CMC) and the branched polysaccharides β-D-glucan (mixed linkage glucan or MLG) and xyloglucan, but displays very poor activity against insoluble, phosphoric acid swollen cellulose (PASC) (Table 2), supporting CelD’s characterization as a broad-spectrum endoglucanase. Time course measurements are provided in Supplemental Fig. S5.

**Table 2:** Specific activity of the CelD catalytic domain against several substrates

	CMC	β-D-glucan	Xyloglucan	PASC
Specific activity $\left(\frac U{\mathrm\mu\;\mathrm{mol}\;\mathrm{enzyme}}\right)$	117.8 ± 5.8	249.4 ± 10.8	191.4 ± 12.8	0.11 ± 0.14

Activities are reported as the mean ± standard deviation in units of $$\mu mol glucose equivalent min{.}^{-1}$$ (U) per μmol enzyme. *CMC* carboxymethylcellulose, *PASC* phosphoric acid swollen cellulose

The enzyme showed no activity against xylan or arabinogalactan or β-glucosidase activity (data not shown). Relative positions of key pyranose binding residue W44, which interacts with the substrate at the − 3 position, and the catalytic E154 position corroborate the lack of β-glucosidase activity (Fig. 2a, b).

We measured kinetic rate parameters for the CelD catalytic domain acting on the soluble cellulase substrate carboxymethylcellulose (CMC) at 39 °C and pH 5.5, the physiological temperature for anaerobic fungi and acidic pH typical of endoglucanase enzymes. Our measured *k*_cat_ and *K*_m_ for *P. finnis* CelD are comparable to those of other fungal endoglucanases but well below those of thermophilic bacterial cellulose degraders like *Thermotoga maritima* and *Clostridium thermocellum* (Table 3).

**Table 3:** Kinetic parameters of *P. finnis* CelD for CMC hydrolysis in comparison to other GH5 family members

Enzyme	*k*_cat_ (s^−1^)	*K*_m_ (g/L CMC)	*k*_cat_/*K*_m_ (L/g/s)	Source
*Piromyces finnis* CelD catalytic domain	6.0 ± 0.58	7.6 ± 2.1	0.8 ± 0.2	This work
*Trichoderma reesei* EglII	1331 ± 37	0.84 ± 0.14	1584	Qin et al. (2008)
*Thermotoga maritima* Cel12A	791	2.1	402	Cheng et al. (2012)
*Penicillium verruculosum* EG2	152	22.8 ± 0.2	6.6 ± 0.1	Merzlov et al. (2015)
*Thermoanaerobacter tengcongensis* MB4 Cel5A	1.94 ± 0.01	1.4 ± 0.1	1.4 ± 0.1	Liang et al. (2011)
*Aspergillus fumigatus* Egl2010	6	2.0 ± 0.6	3.0	Ren (2009)
*Aspergillus nidulans* Egl2010	4	29 ± 8.8	0.1	Ren (2009)
*Fusarium graminearum* Egl2010	14	13 ± 4.8	1.1	Ren (2009)
*Aureobasidium pullulans* SEQ15654	29	10 ± 2.9	2.9	Ren (2009)
*T. reesei* Eg2/Cel5a	4	2.6 ± 0.7	1.5	Ren (2009)
*Gloeophyllum trabeum* SEQ630	6	13 ± 3.9	0.5	Ren (2009)
*Sporotrichum thermophile* SEQ13822	6	3.3 ± 0.6	1.8	Ren (2009)
*Clostridium thermocellum* EngD	30.1	6.5	4.6	Ren (2009)
*Martelella mediterranea* Cel5D	3.5	8.8 ± 0.1	0.4	Dong et al. (2010)

### CelD cellulase kinetics are unperturbed by the addition of N- and C-Terminal dockerins

**Fig. 4:**
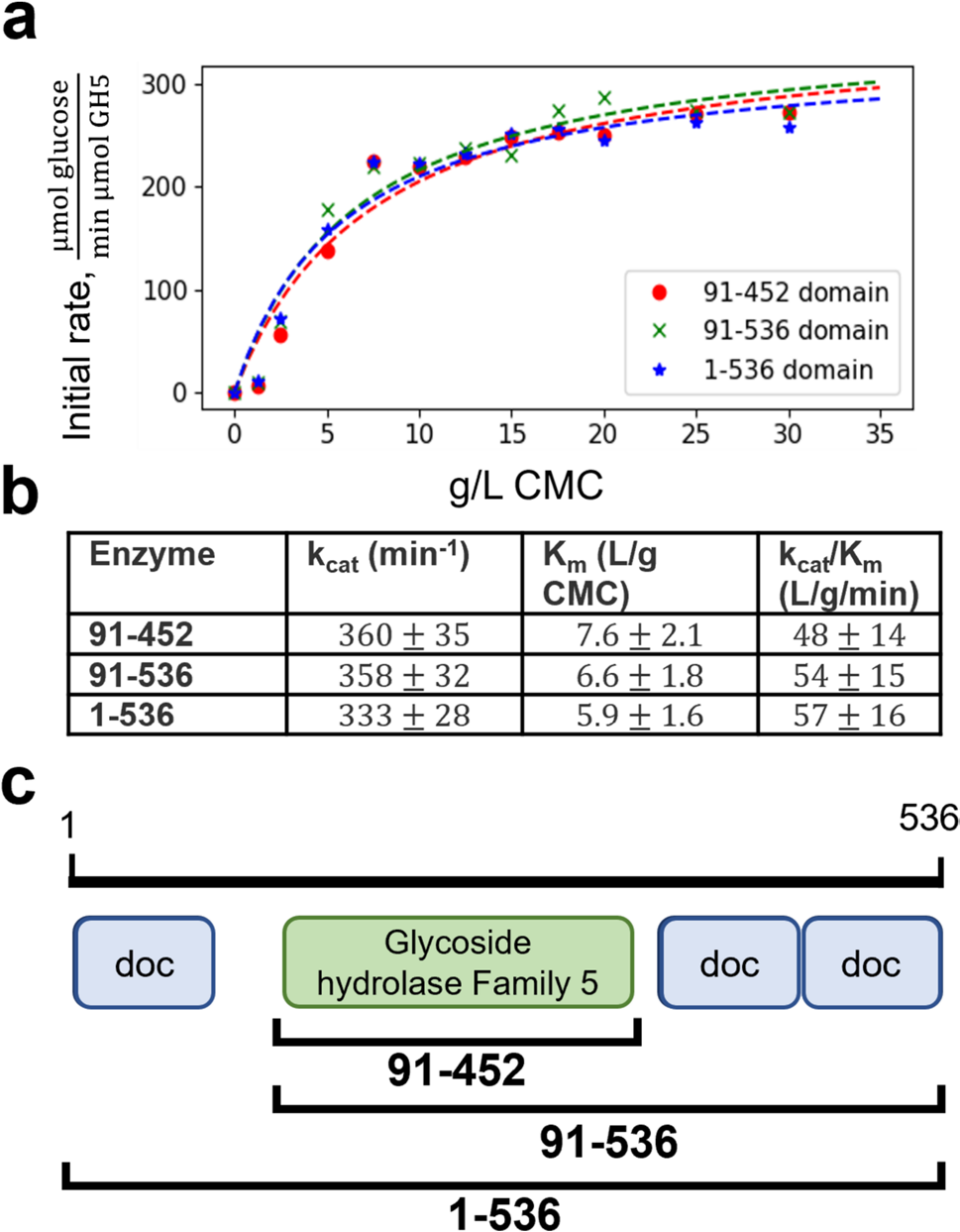
Addition of N- and C-terminal fungal dockerin domains does not affect enzyme kinetics. (**a**) Kinetic parameters for hydrolysis of carboxymethylcellulose (CMC) were fit from initial rate data for the GH5 catalytic domain alone (91–452, red circles) as well as the GH5 with two C-terminal dockerins (91–536, green crosses) and the GH5 with N- and C-terminal dockerins (1–536, blue stars). (**b**) Initial rates for each enzyme at each substrate concentration were taken from time course measurements of released reducing sugar vs time, as quantified by the DNS assay. Fit parameter uncertainties are reported ± one standard deviation from nonlinear least squares fit. (**c**) A diagram showing the relative position of dockerins (abbreviated “doc” for short) relative to the GH5 for tested variants

A key question we sought to answer was whether CelD’s natural C-terminal dockerin domains conferred any catalytic benefit and whether this enzyme could tolerate non-natural dockerin domain fusions, such as one on its N-terminus. True modularity in the construction of catalytic domain–dockerin domain chimeras is highly desirable in building synthetic enzyme systems for applications like lignocellulose valorization. Our results show that CelD’s intrinsic kinetics are unchanged when dockerin domains are fused to the N- or C-terminus of this protein, indicating the natural CelD protein is highly modular and suggesting that the CelD catalytic domain can accommodate other fusion partners (Fig. 4).

## Discussion

This crystal structure represents one of only a few solved structures of anaerobic fungal enzymes and is the second GH5 from *Neocallimastigomycota* to be solved (Tseng et al. 2011), presenting a reference by which to compare other GH5 endoglucanases from *Neocallimastigomycetes* (of which there are 637 unique, annotated sequences, www.mycocosm.jgi.doe.gov), towards identifying GH5 enzymes with desirable properties for industrial bioprocessing. CelD displayed endoglucanase activity against β-1,4-glycosidic bonds in CMC, xyloglucan, and β-D-glucan, and measured kinetic parameters on CMC enabled direct comparison of CelD to other endoglucanases.

Structural features that differentiated the *P. finnis* catalytic domain from other GH5 enzymes were longer loops around the substrate binding site (between *β*_I_/*α*_1_, *β*_IV_/*α*_4_, *α*_5_/*β*_VI_, and *β*_VIII_/*α*_8_) and possessing only one disulfide bridge instead of four present in GH5’s from other fungi (Lee et al. 2002, 2011). Further biochemical studies are needed to investigate the contribution of these loop regions and missing disulfide bonds to enzyme–substrate specificity and thermal stability.

The effect of fungal dockerin domains on the activity of gut fungal enzymes has previously been evaluated in only a few cases with conflicting results. While no change in activity was observed upon removal of the native C-terminal dockerin from a *Piromyces* mannanase at 39 °C (Fanutti et al. 1995), Huang and co-authors (2005) found removal of the native C-terminal double dockerin from *Neocallimastix frontalis* Xyn11A and Xyn11B to increase specific xylanase activity at all temperatures (39–70 °C). We have also found the addition of the C-terminal double dockerin from *P. finnis* CelD to *T. maritima* enzymes Cel5A and XynA to cause insignificant changes to specific enzyme activity at 80 °C (Gilmore et al. 2020).

This lack of consistency suggests the effect of fungal dockerin fusions on catalytic domain activity is context dependent, at least when evaluating enzymes recombinantly produced in *E. coli*. Unfortunately, our attempts to crystallize a construct containing both the catalytic and dockerin domains were unsuccessful, and a structure of a complete, dockerin-containing enzyme from an anaerobic fungus with which to definitively address these questions remains unsolved. Negative effects of dockerin domains on enzymatic activity have previously been tied to a reduction in protein thermostability and melting temperature (Huang et al. 2005). However, it is difficult to decouple potential intrinsic instability of the dockerin domain from the possibility that these domains, which are known to possess several disulfide bonds (Raghothama et al. 2001; Nagy et al. 2007), are misfolded when produced recombinantly in *E. coli*. More efficient disulfide bond formation was shown to have a dramatic impact on the measured enzymatic activity of a non-dockerin-containing *Neocallimastix patriciarum* xylanase, which the authors evaluated by producing the same enzyme in *E. coli* and *Pichia pastoris* (Cheng et al. 2014). Ongoing work investigating dockerin-containing enzymes from their native system will build on these previous results to address this outstanding question of how dockerin domains contribute to enzyme activity and stability.

Anaerobic fungi deploy an array of CAZymes that act in solution and as members of multi-enzyme cellulosomes to rapidly hydrolyze lignocellulose. However, very few enzymes in the vast CAZyme repertoire encoded by anaerobic fungal genomes have been functionally characterized, and as a result, we have little biochemical understanding of how anaerobic fungi excel at degrading biomass, which presents a challenge towards converting anaerobic fungal enzyme systems into useful biotechnologies. By characterizing the atomic-resolution structure and kinetic properties of the *P. finnis* CelD GH5 endoglucanase, we provide additional insight towards gaining biochemical understanding of anaerobic fungal enzyme systems. The kinetic data indicate the domains of CelD are highly modular and can likely be augmented to functionalize this GH5 enzyme with other domains, while the structure presents a platform for rational engineering of this enzyme for higher thermostability or activity criteria.

## Supplementary Information

Below is the link to the electronic supplementary material.

Supplementary file1 (PDF 1819 KB)

## Data Availability

Crystallographic data for structures reported in this article were deposited to the Protein Data Bank (PDB codes 8GHX and 8GHY for the apo and the E154A-mutant complex, respectively). All other relevant data generated and analyzed in this study are included in this article or the supplementary information. Plasmids encoding the enzyme constructs studied in this work are available upon reasonable request.
