# Supplementary material for: Structure and enzymatic characterization of CelD endoglucanase from the anaerobic fungus Piromyces finnis
Source: Appl Microbiol Biotechnol. 2023 Aug 7;107(19):5999–6011. doi: 10.1007/s00253-023-12684-0 (PMC10485095; doi:10.1007/s00253-023-12684-0)
Supplement: Supplementary file 1 — Supplementary file1 (PDF 1819 KB) [file 253_2023_12684_MOESM1_ESM.pdf]

Structure and Enzymatic Characterization of CelD Endoglucanase from the Anaerobic Fungus *Piromyces finnis*

Alexey Dementiev[2*], Stephen P. Lillington[1*], Shiyan Jin[1], Youngchang Kim[2], Robert Jedrzejczak[2], Karolina Michalska[2], Andrzej Joachimiak[2,3], Michelle A. O'Malley[1,4,5]

[1] Department of Chemical Engineering, University of California, Santa Barbara, CA USA

[2] Structural Biology Center, X-ray Science Division, Argonne National Laboratory,

Argonne, IL 60439, USA,

[3] Department of Biochemistry and Molecular Biology, University of Chicago, Chicago,

IL 60637, USA,

[4] Biological Engineering Program, University of California, Santa Barbara, CA USA

[5] Joint BioEnergy Institute (JBEI), Emeryville, CA, 94608, USA

*Equal contributions

Corresponding author: Michelle A. O'Malley, momalley@ucsb.edu

Contents:

Table S1 – Amino acid sequences for CelD constructs characterized in this work

Figure S1 – Multiple sequence alignment of homologous GH5 subfamily 4 enzymes to *Piromyces finnis* CelD
Figure S2 – Structural alignment of homologous GH5 subfamily 4 enzymes to *Piromyces finnis* CelD
Figure S3 - Sequence alignment of fungal cellulases from *Piromyces finnis* (CelD), *Thermoascus aurantiacus* (EngI), *Trichoderma reesei* (EgII), and *Piromyces rhizinflata* (EglA)

Figure S4 - Structural comparison of the fungal cellulase catalytic domains.

Figure S5 – Timecourse measurements of reducing sugar concentration for the CelD catalytic domain against several substrates.

Table S1. Amino acid sequences for CelD constructs characterized in this work.

**Construct**	**Amino acid sequence**	**Predicted Molecular Weight (kDa)**
91-452 (catalytic domain only)	AIRDISSLELIKEMRFGWNLGNTLDAECTSWMDYEKNPIGSETCWG<br>NVKTNEDIFKTLMDNQFNVFRIPTTWTGHIGEAPEYKINEQWMKRV<br>HEIVDYPYKNGAFVILNIHHETWNHAFAETVDEAKVELAQVWKQIA<br>EEFKGYGERLIFEGQNEPRKNGTPVEWNGGDKEGWDVVNAMNAVFL<br>ETVRSSGGNNAKRHLMIPPYAAACNENSFKNFDFPEDDDKVIASVH<br>AYSPYNFALNNGEGAVDKFDASGKNELDWNINLMKKRFVDQGIPMI<br>LGEYGAMNRDNEEERAAWAEYYMEKITALGVPQVWWDNGVFEGEGE<br>RFGLIDRKNLKIVYPSIVAALQKGRGLEVNVLHAIETE	41.2
91-452 E154A	AIRDISSLELIKEMRFGWNLGNTLDAECTSWMDYEKNPIGSETCWG<br>NVKTNEDIFKTLMDNQFNVFRIPTTWTGHIGEAPEYKINEQWMKRV<br>HEIVDYPYKNGAFVILNIHHETWNHAFAETVDEAKVELAQVWKQIA<br>EEFKGYGERLIFEGQN**A**PRKNGTPVEWNGGDKEGWDVVNAMNAVFL<br>ETVRSSGGNNAKRHLMIPPYAAACNENSFKNFDFPEDDDKVIASVH<br>AYSPYNFALNNGEGAVDKFDASGKNELDWNINLMKKRFVDQGIPMI<br>LGEYGAMNRDNEEERAAWAEYYMEKITALGVPQVWWDNGVFEGEGE<br>RFGLIDRKNLKIVYPSIVAALQKGRGLEVNVLHAIETE	41.1
91-536 (catalytic domain + C-terminal double dockerin. Dockerins highlighted in bold)	AIRDISSLELIKEMRFGWNLGNTLDAECTSWMDYEKNPIGSETCWG<br>NVKTNEDIFKTLMDNQFNVFRIPTTWTGHIGEAPEYKINEQWMKRV<br>HEIVDYPYKNGAFVILNIHHETWNHAFAETVDEAKVELAQVWKQIA<br>EEFKGYGERLIFEGQNEPRKNGTPVEWNGGDKEGWDVVNAMNAVFL<br>ETVRSSGGNNAKRHLMIPPYAAACNENSFKNFDFPEDDDKVIASVH<br>AYSPYNFALNNGEGAVDKFDASGKNELDWNINLMKKRFVDQGIPMI<br>LGEYGAMNRDNEEERAAWAEYYMEKITALGVPQVWWDNGVFEGEGE<br>RFGLIDRKNLKIVYPSIVAALQKGRGLEVNVLHAIETEPTE**CWSLK**<br>**YGYECCSPNNTRVVVTDESGKWGVENSDWC**GIVDSKDK**CWSIPFGY**<br>**KCCDHCKVLLTDESGKWGELNGEWCGIDTTKC**K	51.0
1-536 (N-terminal dockerin + catalytic domain + C-terminal double dockerin. Dockerins highlighted in bold.)	MRILNVLSLTGLVIAGTNALD**CWANKLGYDCCKTSKEVYEDVEGKW**<br>**GVENGNWC**GIVEEEPTTTVVELVEEPTEPAVDVEEPTEPVELEPIR<br>DISSLELIKEMRFGWNLGNTLDAECTSWMDYEKNPIGSETCWGNVK<br>TNEDIFKTLMDNQFNVFRIPTTWTGHIGEAPEYKINEQWMKRVHEI<br>VDYPYKNGAFVILNIHHETWNHAFAETVDEAKVELAQVWKQIAEEF<br>KGYGERLIFEGQNEPRKNGTPVEWNGGDKEGWDVVNAMNAVFLETV<br>RSSGGNNAKRHLMIPPYAAACNENSFKNFDFPEDDDKVIASVHAYS<br>PYNFALNNGEGAVDKFDASGKNELDWNINLMKKRFVDQGIPMILGE<br>YGAMNRDNEEERAAWAEYYMEKITALGVPQVWWDNGVFEGEGERFG<br>LIDRKNLKIVYPSIVAALQKGRGLEVNVLHAIETEPTE**CWSLKYGY**<br>**ECCSPNNTRVVVTDESGKWGVENSDWC**GIVDSKDK**CWSIPFGYKCC**<br>**DHCKVLLTDESGKWGELNGEWCGIDTTKC**K	60.8

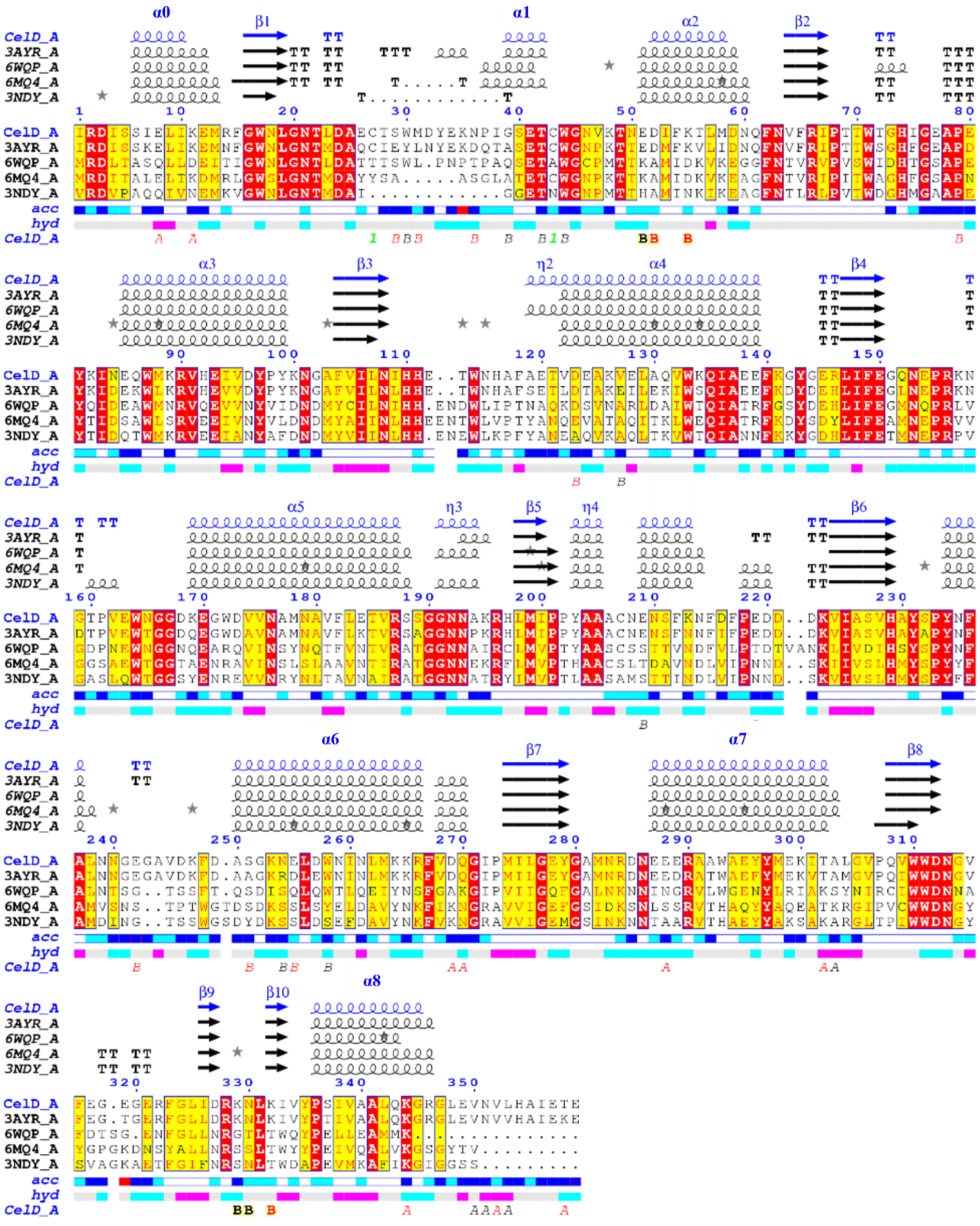

Figure S1. Sequence alignment of homologous GH5_4 enzymes with known structures. Strictly conserved residues are shown in red block and chemically similar residues shown in yellow block. “acc” represents the solvent accessibility and “hyd” the hydropathy of the sequence. 6WQP contains two mutations from the wild-type enzyme sequence (Uniprot Accession no. D4LAX7), E154Q and E278Q in the multiple sequence alignment. Figure generated with Endscript (Robert and Gouet 2014).

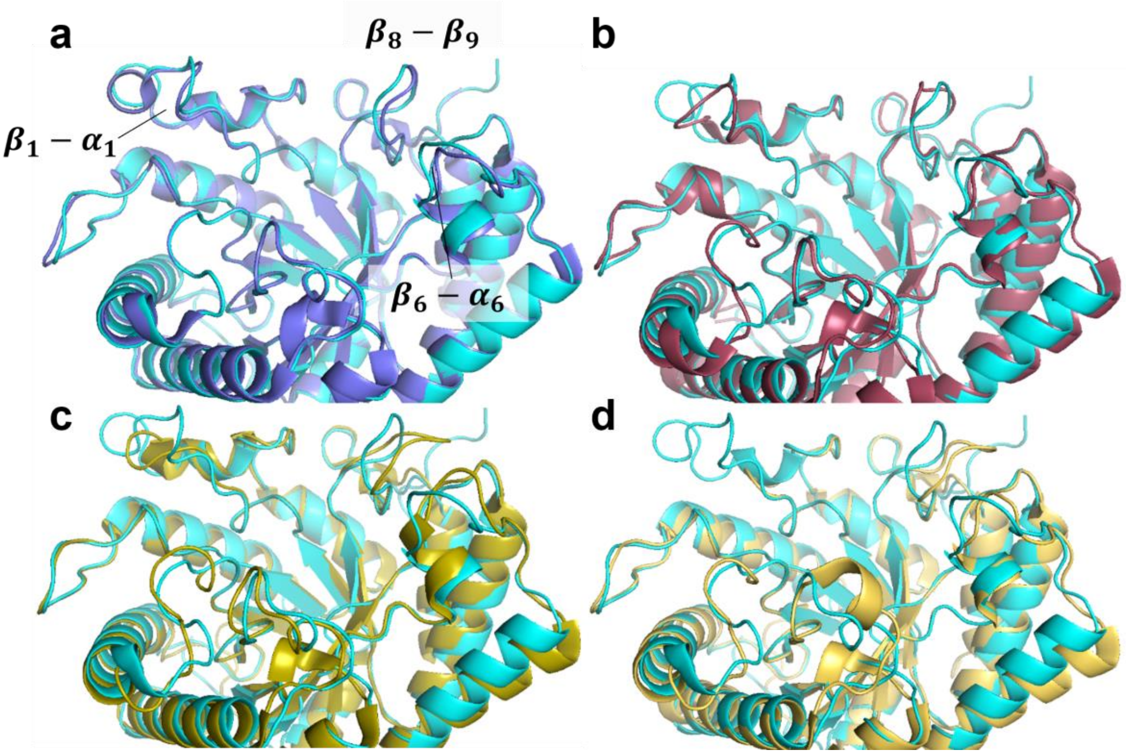

Figure S2. Structural alignment of CelD (cyan) to 3AYR (a), 6WQP (b), 6MQ4 (c), and 3NDZ (d). The loops exhibiting the greatest structural diversity are annotated and CelD is shown in the same orientation in each panel.

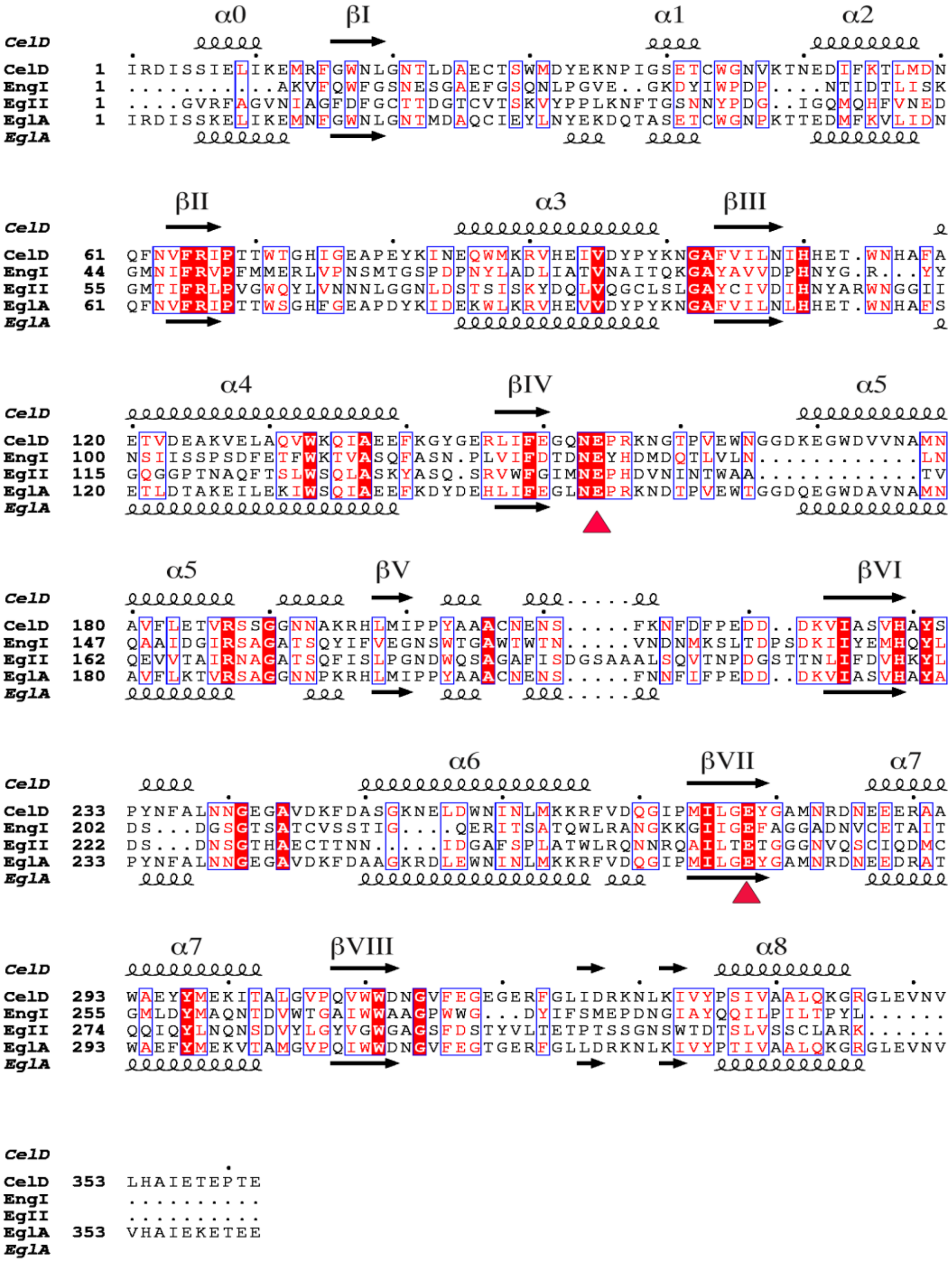

Figure S3. Sequence alignment of fungal cellulases from *Piromyces finnis* (CelD), *Thermoascus aurantiacus* (EngI), *Trichoderma reesei* (EgII), and *Piromyces rhizinflata* (EglA). Strictly conserved residues are shown in red block, and chemically similar residues in red text. The residue numbering is shown on the left for each catalytic domain. Dashed lines indicate deletions. The acid/base and nucleophile residues are indicated below by red triangles. The secondary structure elements of CelD and EglA are shown above and below the alignment, respectively. The sequence alignment revealed close homology between CelD and EglA cellulases (82% amino acid residue identity). The figure was generated with ESPript (http://espript.ibcp.fr) (Robert and Gouet 2014).

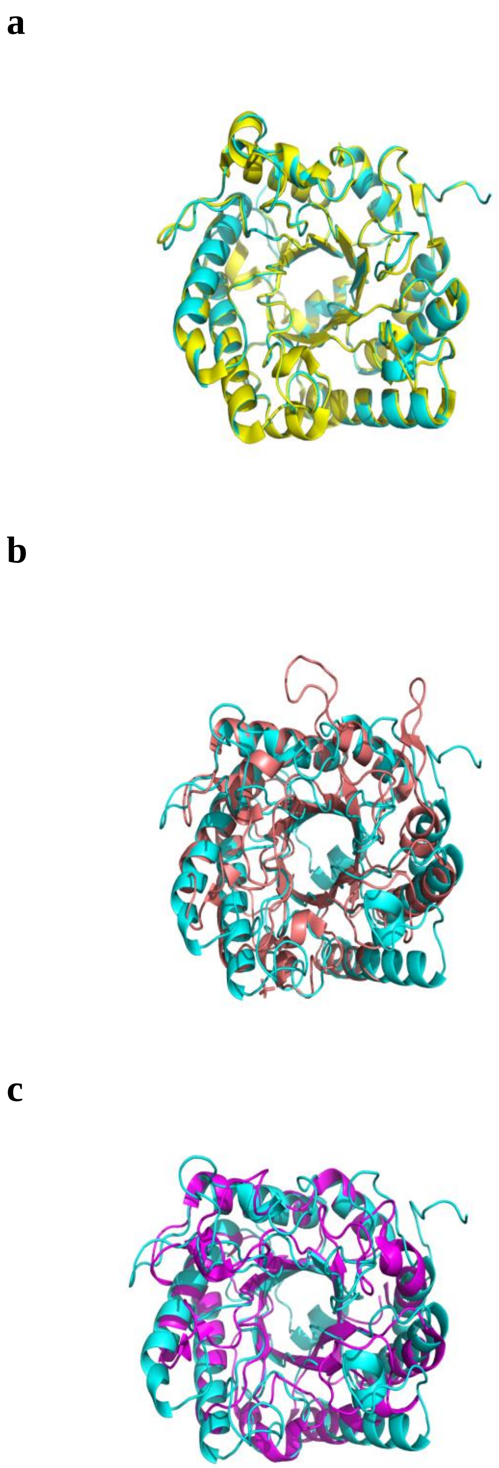

Figure S4. Structural comparison of the fungal cellulase catalytic domains. (A, B, C) Superposition of the CelD structure reported in this study (cyan ribbon) and the catalytic domain of EglA (PDB code 3AYR, yellow ribbon), EgII (PDB code 3QR3, red ribbon), and EngI (PDB code 1GZJ, magenta ribbon).

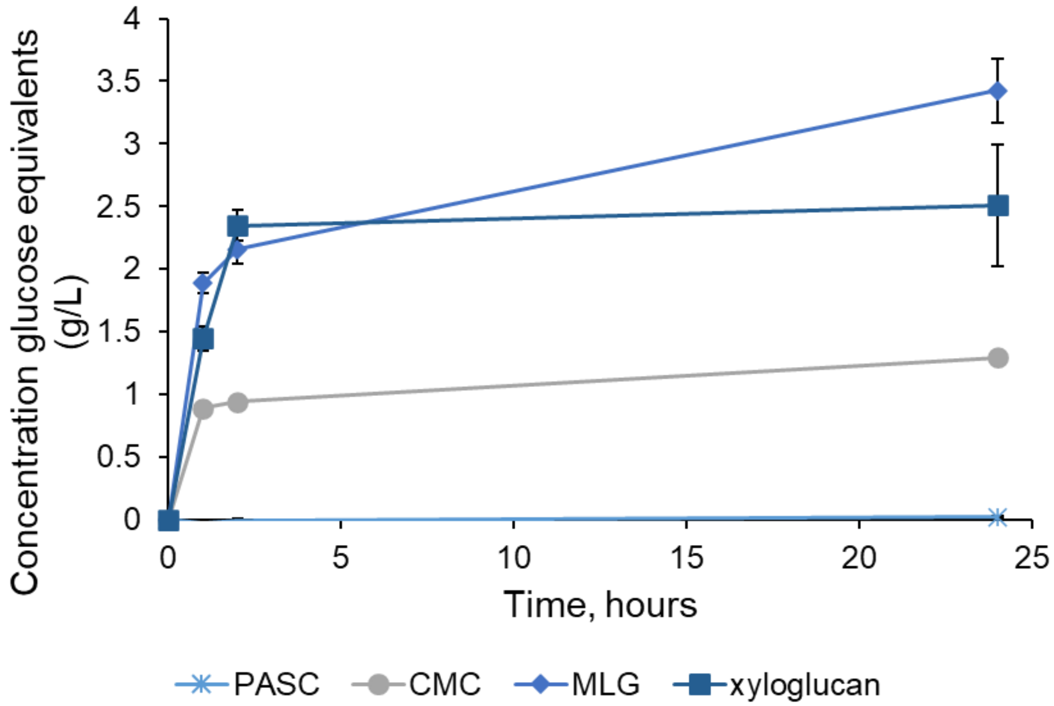

Figure S5. Timecourse measurement of reducing sugar concentration for CelD catalytic domain against (hemi)cellulosic substrates. Data are presented as the mean ± standard deviation of technical triplicates. PASC: phosphoric acid swollen cellulose; CMC: carboxymethylcellulose; MLG: mixed linkage glucan (β-D-glucan).

Supplementary References

Robert X, Gouet P (2014) Deciphering key features in protein structures with the new ENDscript server. Nucleic Acids Res 42:W320–W324. https://doi.org/10.1093/nar/gku316
